# Supplementary material for: Preoperative Biomarkers and Survival in Chinese Breast Cancer Patients with HIV: A Propensity-Score-Matched-Cohort Study
Source: Viruses. 2023 Jun 30;15(7):1490. doi: 10.3390/v15071490 (PMC10383696; doi:10.3390/v15071490)
Supplement: Supplementary file 1 [file viruses-15-01490-s001.zip › viruses-2399472-supplementary.pdf]

Supplementary Materials

Table S1: Diagnostic value of different prognosis biomarkers calculated by Max rank statistic.

	Overall Survival			Progression Free Survival		
	cut off value	statistics	Log-rank	cut off value	statistics	Log-rank
Neutrophil-to-lymphocyte ratio	0.88	1.90	0.21	1.50	1.79	0.08
Lymphocyte-to-monocyte ratio	3.59	1.72	0.06	3.59	2.13	0.02
Platelet-to-lymphocyte ratio	98.17	1.91	0.09	98.17	2.36	0.03
CD4	550.00	1.16	0.35	336.70	1.55	0.13
CD8	758.00	2.26	0.04	662.70	2.63	<0.01
CD4/CD8	0.48	1.83	0.08	0.29	1.99	0.06
CD3	1033.80	1.50	0.08	1033.80	2.29	0.01

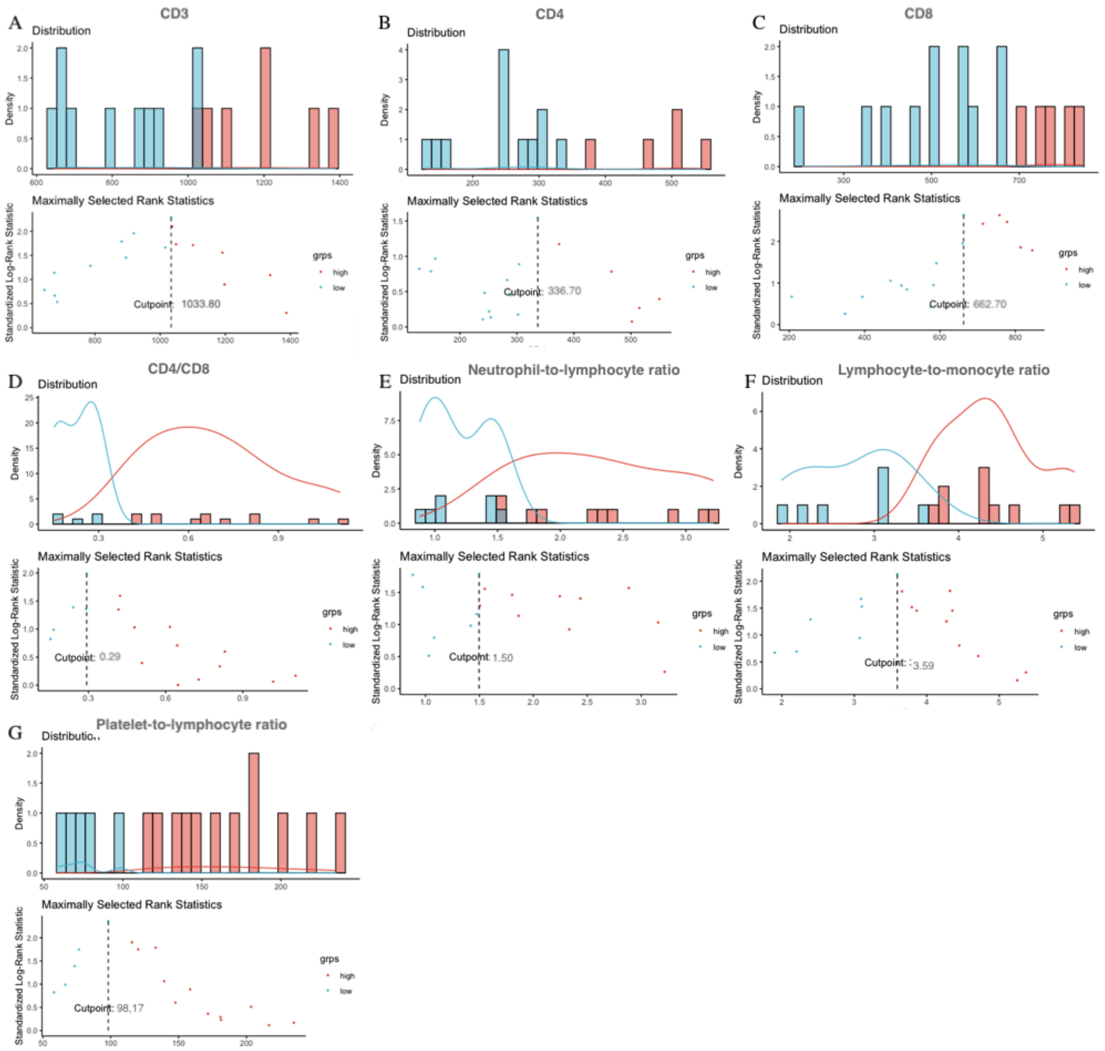

Figure S1. Distribution plot of different preoperative biomarkers for progression free survival in breast cancer patients with HIV. (A) CD3; (B) CD4; (C)CD8; (D) CD4/CD8 ratio; (E) neutrophil-to-lymphocyte ratio; (F) lymphocyte-to-monocyte ratio; (G) platelet-to-lymphocyte ratio.

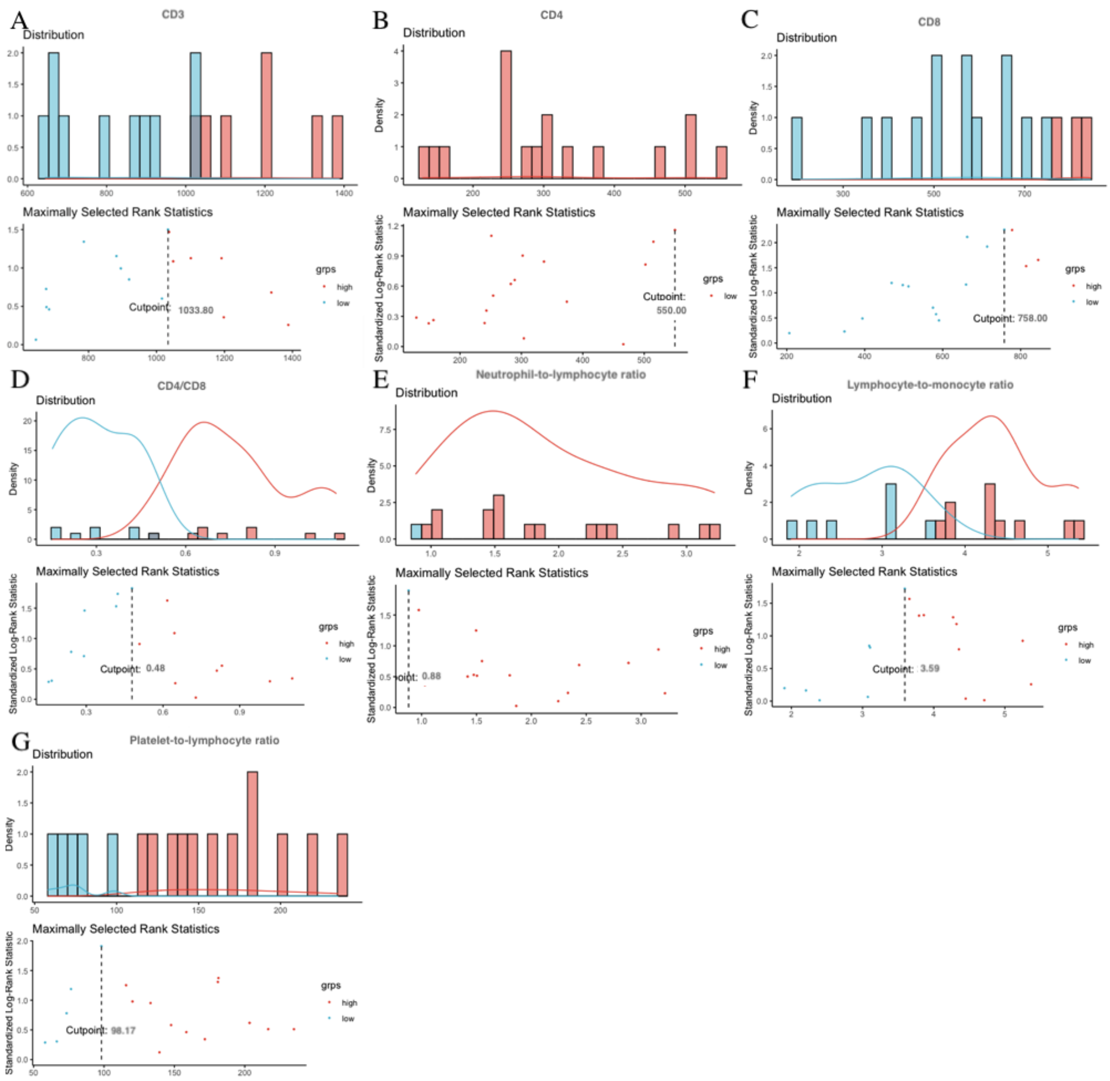

Figure S2. Distribution plot of different preoperative biomarkers for overall survival in breast cancer patients with HIV. (A) CD3; (B) CD4; (C)CD8; (D) CD4/CD8 ratio; (E) neutrophil-to-lymphocyte ratio; (F) lymphocyte-to-monocyte ratio; (G) platelet-to-lymphocyte ratio.
